# Post-traumatic cilia remaining inert in the anterior chamber for 50 years: a case report

**DOI:** 10.1186/1752-1947-5-527

**Published:** 2011-10-26

**Authors:** Zuleyha Yalniz-Akkaya

**Affiliations:** 1 Private Practice, Ataturk Blv. 1235 Sk No:1A Umut Eye Center, Aksaray, TR68100, Turkey

## Abstract

**Introduction:**

The present report concerns what is, to the best of our knowledge, the first case of post-traumatic cilia that has remained inert for approximately 50 years after its inoculation into the eye.

**Case presentation:**

A 69-year-old Caucasian woman whose right eye had been struck by a dining fork approximately 50 years earlier was examined on presentation two years ago. In her right eye, both uncorrected and best-corrected visual acuities were 0.1 (in decimal notation). Along with a nuclear cataract, a straight linear extension was found extending beneath the iris at the nine o'clock position reaching the center of the pupil, which appeared to be a cilium measuring 7 mm. After the removal of the cilia, an uncomplicated phacoemulsification was performed and a posterior chamber intra-ocular lens was implanted. Her post-operative course was uneventful, and visual acuity remained 1.0 for the 22-month follow-up period.

**Conclusions:**

Intra-ocular cilia can be tolerated for as long as 50 years without causing any ocular reaction.

## Introduction

Cilia in the anterior chamber (AC) constitute a relatively small portion of intra-ocular foreign bodies [1]. The route of intra-ocular access can be via penetrating injury [1-5] or ocular surgery [6]. While some cases are symptomatic [1,3,7,8], some remain asymptomatic [1,2,6,9,10] for years.

## Case presentation

A 69-year-old Caucasian woman with decreased vision in her right eye was examined on presentation two years ago. This was her first ophthalmic examination since birth.

**Figure 1:**
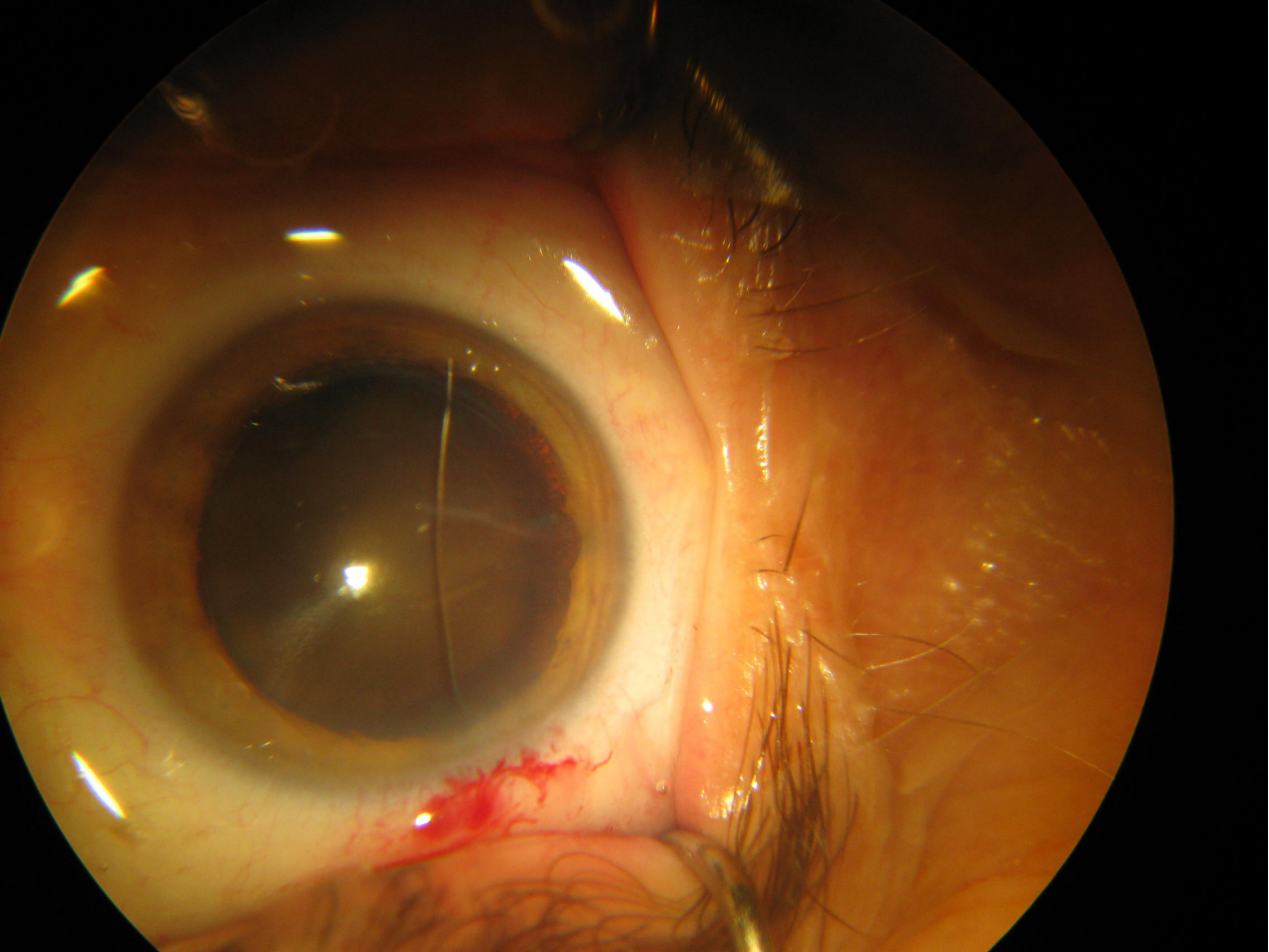
**Cilia in the anterior chamber**. This image was taken intra-operatively.

**Figure 2:**
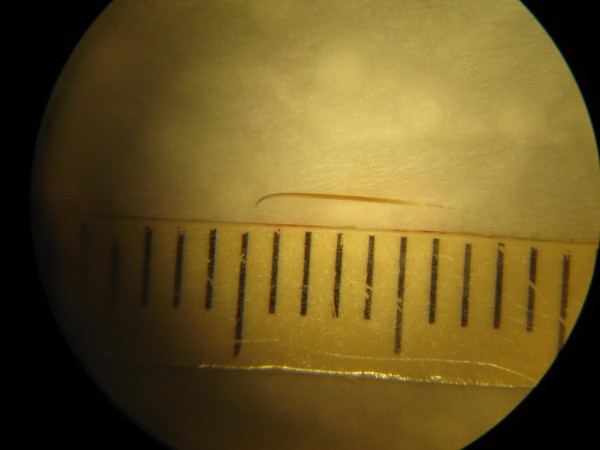
**Cilium removed from the eye**. A well preserved cilium survived in the aqueous environment for approximately 50 years.

**Figure 3:**
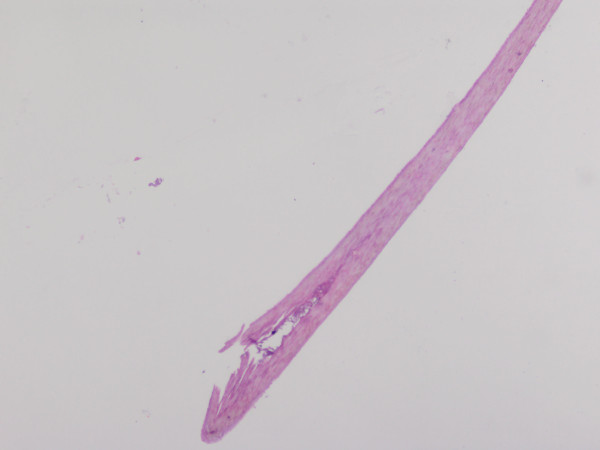
**Histopathological picture of the cilium (×10, hemtaoxilyn and eosin stain)**.

In her right eye, both her uncorrected (UCVA) and best-corrected visual acuities (BCVA) were 0.1 (in decimal notation). The intra-ocular pressure (IOP) measured by Goldmann applanation tonometer was 14 mmHg. Hardly noticeable temporal paracentral corneal opacity and subtle irregularity of the temporal pupillary margin was noted. In the AC, a straight linear extension extending from behind the iris at the nine o'clock position, reaching the center of the pupil and resembling cilia was visible (Figure 1, Figure 2, Figure 3). The anterior chamber was quiet with no cells or flare and no posterior synechia. With gonioscopy, no anterior synechia or second cilia were noted. Although evidence supported previous injury, she strongly denied any ocular trauma. Because of her nuclear cataract, we admitted her cautiously for cataract surgery, and were prepared for unexpected intra-operative findings.

After filling the AC with an ophthalmic viscosurgical device, the extension was mobilized using capsule forceps and appeared to be longer than the visible portion and half-hidden under the temporal iris. After the extraction, it appeared to be a 7 mm-long cilium, the nature of which was confirmed by pathological examination. After the removal of the cilium, an uncomplicated phacoemulsification and +21.00D posterior chamber intra-ocular lens (Ocuflex, Ocu-Ease Optical Products Inc., Pinole, Canada) implantation was performed. At the first post-operative examination, while again evaluating the trauma history, one of her daughters remembered that approximately 50 years earlier our patient's eye had been struck by her little girl with a dining fork, but no medical care was sought at the time. Ofloxacin (Exocin, Allergan Inc., Irvin, USA) was used for one week and Dexamethasone Sodium Phosphate (Maxidex, Alcon Laboratories Inc., Texas, USA) and Ketorolac tromethamine (Acular, Allergan Inc., Irvin, USA) were used for one month. Her post-operative course was uneventful and visual acuity remained 1.0 for 18 months.

In her other eye, both UCVA and BCVA were 0.3. The IOP was 14 mmHg. The cornea was clear, AC was normal, pupil was regular and central, and a nuclear cataract was present. This eye also underwent an uncomplicated phacoemulsification and posterior chamber intra-ocular lens implantation followed by stable post-operative course with BCVA of 1.0.

## Discussion

Cilia can enter into the eye either as a result of penetrating surgery [3,6] or penetrating injury [1-3,5,7,9]. Post-traumatic intra-ocular cilia events comprise a small portion (0.4%) of all intra-ocular foreign bodies [1]. Cilia can be entrapped in the cornea, AC, posterior chamber, lens, vitreous, or retina or can migrate into the eye [3,4,6,7,9-12]. Anterior chamber cilia account for 45% of all intra-ocular ciliae [1].

The response of the eye to the intra-ocular cilia is unpredictable and variable. In the early post-traumatic or post-surgical course, both infection and inflammation can cause a severe ocular reaction. Intra-ocular cilia can be associated with corneal edema, corneal graft rejection, granulomatous and non-granulomatous iridocyclitis, cyst formation, lens abscess vitreous traction, retinal detachment and endophthalmitis [1,4,7,8]. Although cilia may remain inert for many years, exacerbation with delayed inflammatory reactions of various severity may occur, ending with blindness [7].

A literature review revealed that cilia entrapped in the AC can sometimes cause inflammation [3,8] and can sometimes remain innocuous [2,9,11,12]. In the literature, there is a report of silent cilia existing in the AC for 33 years [12]. To the best of our knowledge, our report is the first case of post-traumatic cilia that has remained silent for approximately 50 years. The asymptomatic course of intra-ocular cilia is related to its relatively inert nature compared to other organic materials and the immune privileged feature of the eye [7]. Based on this fact, some practitioners prefer observation in asymptomatic cases [2,9], while others prefer surgical intervention to eliminate the potential of devastating endophthalmitis [6-8].

## Conclusions

In spite of their organic nature, intraocular ciliae can be tolerated for many years without causing any ocular reaction; however, the potential for late severe reaction still exists and makes management controversial.

## Consent

Written informed consent was obtained from the patient for publication of this case report and any accompanying images. A copy of the written consent is available for review by the Editor-in-Chief of this journal.

## Competing interests

The authors declare that they have no competing interests.
